# Effectiveness of nutritional support for clinical outcomes in gastric cancer patients: A meta-analysis of randomized controlled trials

**DOI:** 10.1515/med-2024-1023

**Published:** 2024-09-04

**Authors:** Juping Zhang, Qian Kong, Jibo Zhang, Jun Guo

**Affiliations:** Department of Oncology, Xingtai People’s Hospital, Xingtai, 054000, China; Department of Oncology, Xingtai People’s Hospital, 818 Xiangdu North Road, Xingtai, 054000, China

**Keywords:** gastric cancer, nutritional support, enteral nutrition, immunonutrition, parenteral nutrition, meta-analysis

## Abstract

**Background:**

Gastric cancer (GC) is a leading cause of cancer-related morbidity and mortality globally. This meta-analysis was conducted to assess the impact of nutritional interventions on clinical outcomes in GC patients.

**Methods:**

Comprehensive search was conducted across four medical databases to identify randomized controlled trials (RCTs) that examined nutritional interventions in GC patients. The outcomes assessed included hospitalization duration, nutritional status, immune function, and complications.

**Results:**

A total of 11 studies were included. Enteral nutrition (EN) significantly reduce hospital stay duration compared to no nutritional intervention (SMD = −1.22, 95% CI [−1.72, −0.73], *P* < 0.001) and parenteral nutrition (PN) (SMD = −1.30, 95% CI [−1.78, −0.82], *P* < 0.001), but showed no significant difference compared to immunonutrition (IN). EN also improved nutritional status, indicated by higher albumin prealbumin levels, and improved immune function by elevating CD4+ levels (SMD = 1.09, 95% CI [0.61, 1.57], *P* < 0.001). However, IN showed superior effects on immunoglobulin levels (IgG and IgM). No significant differences were observed in complication rates among EN, IN, and PN interventions.

**Conclusion:**

Nutritional support, particularly EN and IN, can significantly improve hospitalization outcomes, nutritional status, and immune function. Customizing interventions according to patient requirements can optimize therapeutic outcomes, highlighting the need for further research in this area.

## 1 Introduction

Gastric cancer (GC) presents a significant challenge in the field of oncology and is consistently ranked as one of the most commonly diagnosed malignancies worldwide. With approximately one million new cases reported each year, it is a major global health concern [1]. Its prominence is not merely due to its high incidence, but also its substantial contribution to cancer-related morbidity and mortality [2].

The complex pathophysiology of GC leads to a variety of symptoms, such as impaired oral intake, diminished nutrient absorption, and metabolic imbalances, all of which collectively predispose patients to malnutrition [3,4]. The effects of malnutrition in GC patients are far-reaching, influencing treatment outcomes, modulating therapy tolerance, and ultimately affecting survival rates [4].

Various factors contribute to the development of GC. Notably, *Helicobacter pylori* infection, specific dietary inclinations, smoking, and genetic markers emerge as predominant contributors [5]. However, the importance of nutrition in managing GC is often overlooked. As the disease progresses, patients experience a range of symptoms, from mild dysphagia and early satiety to more severe nausea and vomiting. These symptoms, combined with the catabolic nature of malignancies, often lead to significant weight loss, muscle wasting, and reduced physical function [6]. These nutritional challenges not only impact a patient’s quality of life but also pose significant barriers to effective therapeutic interventions [7].

Recognizing the profound implications of nutritional deficits, a wide spectrum of interventional strategies has been conceptualized over the years [8]. These range from dietary guidance and oral supplements to advanced methods like enteral nutrition (EN) and parenteral nutrition (PN) [8,9]. Recent evidence suggests that these nutritional interventions can improve treatment efficacy, decrease hospital stays and post-operative complications, and enhance overall quality of life [8]. Although some studies endorse the effects of these interventions, others express doubts, citing inconclusive or minimal effects [10–12]. These discrepancies indicate the urgent need for a thorough and critical evaluation of existing literature.

Motivated by this imperative, we undertook this meta-analysis. Our main goal is to scrupulously summarize evidence from randomized controlled trials (RCTs), focusing on the nexus between nutritional support and clinical outcomes in GC patients.

## 2 Methods

The meta-analysis was conducted following the Preferred Reporting Items for Systematic Reviews and Meta-Analyses (PRISMA) statement. The study protocol was registered online in the International Prospective Register Systematic Reviews (PROSPERO number: CRD42024558364).

### 2.1 Literature search

A comprehensive search was carried out across multiple medical databases, including PubMed, Embase, Cochrane, and Web of Science, to identify relevant RCTs published from the inception of each database to August 17, 2023. The search strategy utilized a combination of relevant Medical Subject Headings terms and keywords, such as “gastric cancer,” “nutritional support,” “randomized controlled trial” and their synonyms. The detailed search strategy is summarized in Table S1.

### 2.2 Study selection criteria

Studies were eligible if they met the following criteria: (1) RCT design; (2) participants diagnosed with GC; (3) intervention group received nutritional support while the control group underwent standard treatment without nutritional support; (4) reported relevant outcomes such as hospitalization duration, indicators related to nutritional status and immune function, and complications (including gastrointestinal adverse reactions, surgical site infections, anastomotic leakage, and pulmonary infections); and (5) article published in English and Chinese. For the purposes of this study, “immunonutrition” (IN) was defined as enteral feeding routes that include immunomodulating nutrients such as arginine, glutamine, omega-3 fatty acids, and nucleotides. Studies were excluded if they were non-RCT designs, duplicates, reviews, editorials, letters, guidelines, case reports/series, or lacked complete or necessary outcome reporting. Study selection was independently undertaken by two authors, and any discrepancies between these two authors were resolved by a third author.

### 2.3 Data extraction

Data extraction from the selected studies was independently performed by two authors, including the first author’s name, publication year, country, sample sizes, and intervention details. Risk of bias for RCTs was assessed using the Cochrane Risk of Bias tool.

### 2.4 Data analysis

Meta-analysis was performed using RevMan 5.3 software (Cochrane Collaboration, Oxford, UK). The data were presented as mean with standard deviation. Standard mean differences (SMD) with 95% confidence intervals (CI) were used to estimate the effect sizes for continuous variables, while the binomial variables were presented as risk ratio with 95% CI. Heterogeneity in the effect estimate was determined by the Chi-square test, and the inconsistency was quantified using the *I* ^2^ statistics. The choice between random effects (*I* ^2^ > 50%) or fixed-effects (*I* ^2^ ≤ 50%) model was based on the significance heterogeneity. The statistical significance of the pooled estimates was determined using *Z*-test, with a *P* value of <0.05 considered as statistically significant. Publication bias was assessed using funnel plots.

## 3 Results

### 3.1 Literature search and study characteristics

**Figure 1:**
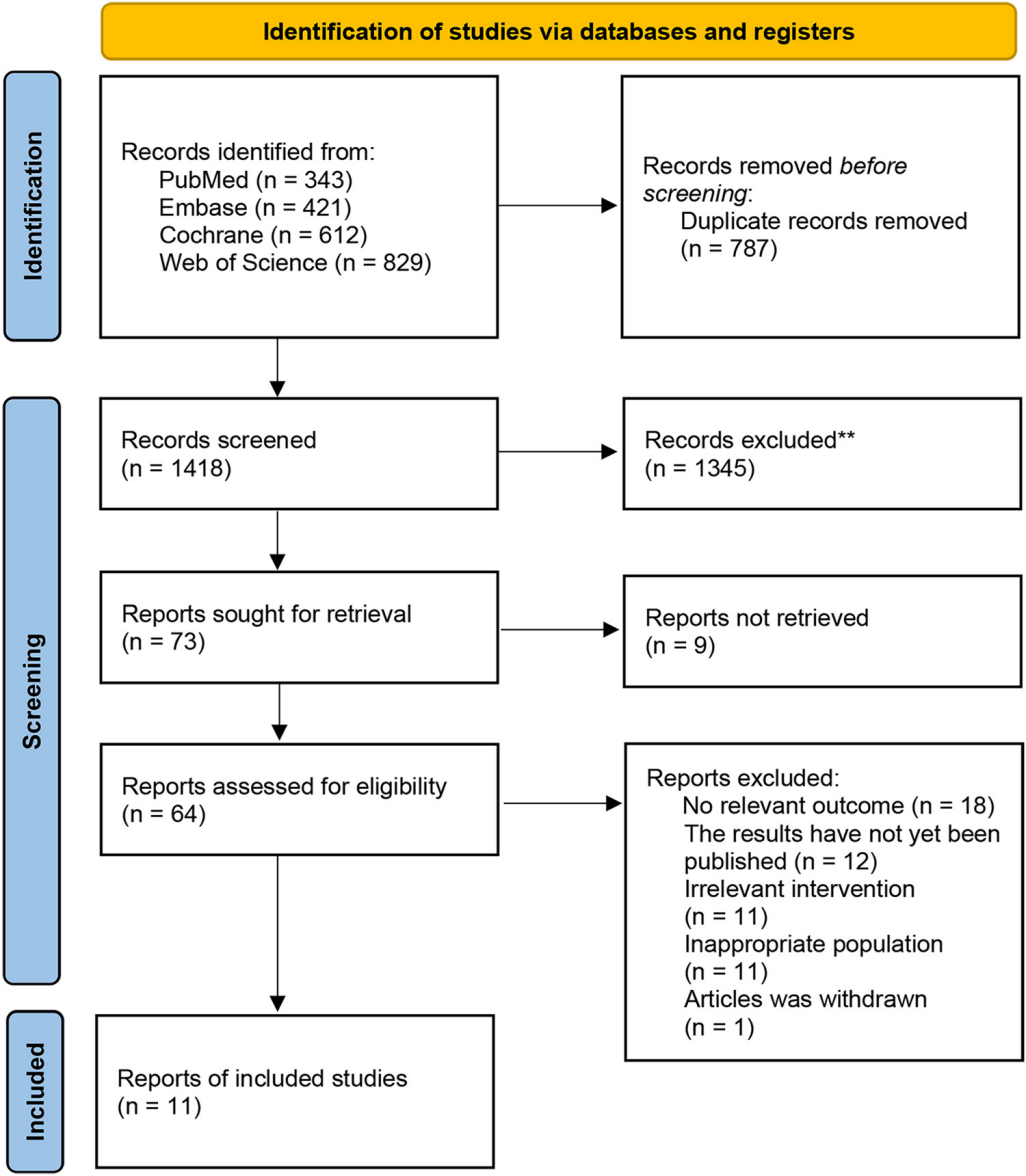
PRISMA flow diagram.

The literature search and study selection process are summarized in Figure 1. A total of 2,205 articles were retrieved – of which 787 were duplicates and 1,345 irrelevant studies were discarded. From 73 screened records, 62 were excluded (of which 9 were not found in full text). Finally, 11 studies including 1,597 GC patients were included for the meta-analysis [9,13–22]. The detailed study characteristics are retrieved in Table 1.

**Table 1:** Characteristics of included studies

First author, year	Country	Patient type	TNM stage	Concurrent therapy	Intervention/control	Sample size (intervention/control)	Mean age (intervention/control)	Time of intervention
Liu, 2011	China	Surgical only	IIb; III; IV	None	Gln: glutamine-enhanced EN	28/28/28	Gln: 71.5 ± 6.1	7 days after surgery
					EN: conventional EN		EN: 74.1 ± 9.3	
					PN: parenteral nutrition		PN: 70.2 ± 8.1	
Fujitani, 2012	Japan	Surgical only	T1–T4; N0–N3	None	Intervention: immunonutrition	127/117	Intervention: 64 (26–78)	5 days before surgery
					Control: regular diet		Control: 65 (30–79)	
Liu, 2012	China	Surgical Only	NR	None	EN + Gln: immune-enhanced EN (EN + Gln)	28/24/26	EN + Gln: 57.3 ± 7.1	Within 48 h after the surgery for 7 days
					EN: standard EN		EN: 58.4 ± 6.3	
					Control: standard nutritional support		Control: 56.2 ± 6.7	
Marano, 2013	Italy	Surgical only	T1–T4; N0–N3	None	Intervention: enteral immunoenriched nutrition	54/55	Intervention: 66.6 (55–78)	From 6 h after surgery until the seventh postoperative day
					Control: standard EN		Control: 65.1 (49–83)	
Chen, 2014	China	Surgical only	I; II; III	None	Intervention: early EN	50/50	Intervention: 59.5 ± 6.6	7 days after surgery
					Control: PN		Control: 59.1 ± 5.9	
Pu, 2014	China	Surgical only	NR	None	Intervention: early EN with long peptide mixture	35/35	Intervention: 60.5 ± 9.3	Within 24 h after the surgery for 6 days
					Control: early EN with glucose saline		Control: 61.3 ± 10.2	
Li, 2015(1)	China	Surgical only	NR	None	Intervention: early EN	200/200	Intervention: 60.8 ± 5.9	Postoperative for 7 days
					Control: PN		Control: 56.0 ± 7.6	
Li, 2015(2)	China	Surgical only	NR	None	Intervention: early EN	150/150	Intervention: 59.2 ± 9.7	From postoperative day 2 to postoperative day 7
					Control: conventional perioperative treatment		Control: 60.4 ± 9.2	
Cui, 2017	China	Surgical only	NR	Postoperative chemotherapy	Intervention: ONS	13/10	Intervention: 58.2 ± 17.5	From first day after discharge from surgery and lasts for 90 days
					Control: dietary guidance		Control: 67.9 ± 8.5	
Ida, 2017	Japan	Surgical only	Clinical T1–T4a and M0 disease	Patients diagnosed with pathological stage II or III disease received adjuvant chemotherapy using S-1. Adjuvant therapy was initiated within 6 weeks after surgery and continued for 1 year	Intervention: ONS (EPA + standard diet)	63/60	Intervention: 65.1 (31–79)	7 days before and 21 days after surgery
					Control: Standard diet		Control: 65.6 (30–80)	
He, 2022	China	Surgical only	T2-4aN0-3M0	None	Intervention: ONS	31/35	Intervention: 63.2 ± 12.0	From the first day after surgery to the fifth day
					Control: dietary advice		Control: 60.5 ± 9.4	

Gln, glutamine; EN, enteral nutrition; PN: parenteral nutrition; EPA, eicosapentaenoic acid; ONS: oral nutritional supplement; NR: not reported.

### 3.2 Study quality assessment

**Figure 2:**
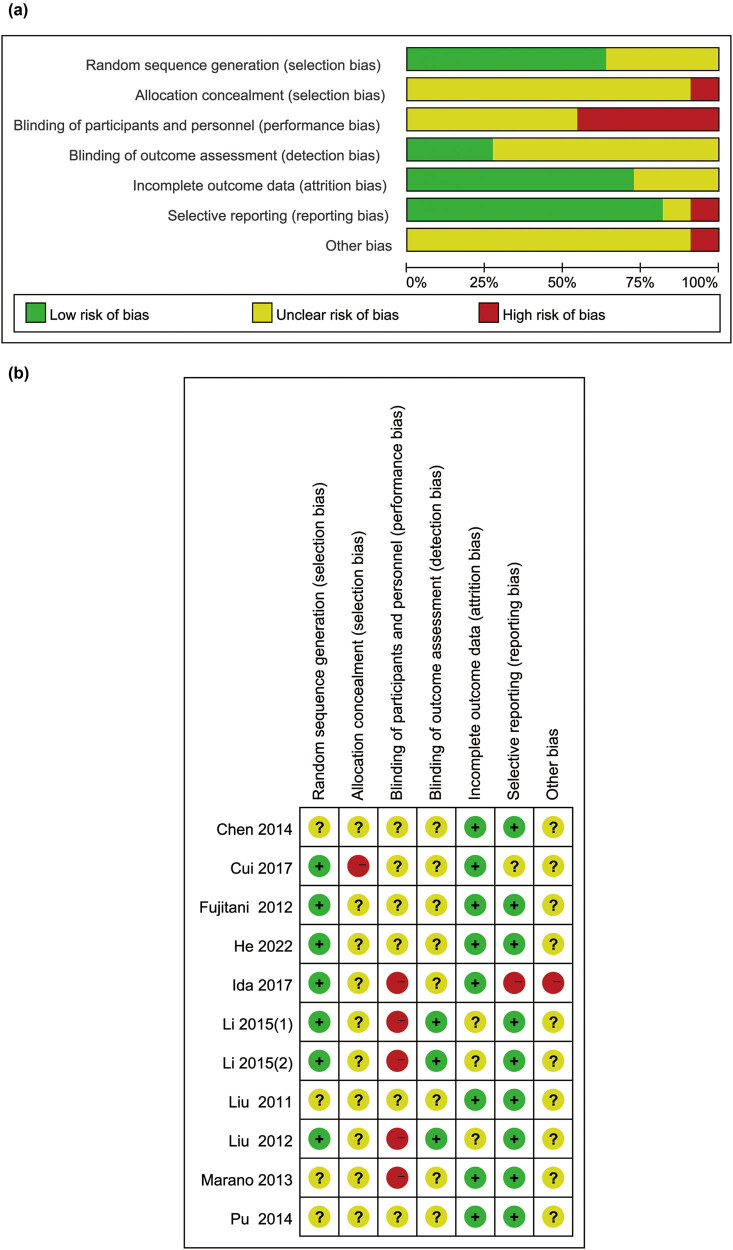
Risk of bias assessment: (a) risk of bias graph and (b) risk of bias summary.

The quality of included studies was assessed according to risk of bias (Figure 2). Among them, seven studies performed appropriate randomization procedures, and four studies reported no or unclear information about randomization procedures. Information on allocation concealment and blinding was unclear or has high risk in all studies, due to the nature of nutritional interventions, and the fact that subjects might be aware of their intervention. Besides, most studies provided adequate outcome data and adequate reporting of results because they reported the same results as described in Section 2.

### 3.3 Duration of hospital stay and time to first flatus

**Figure 3:**
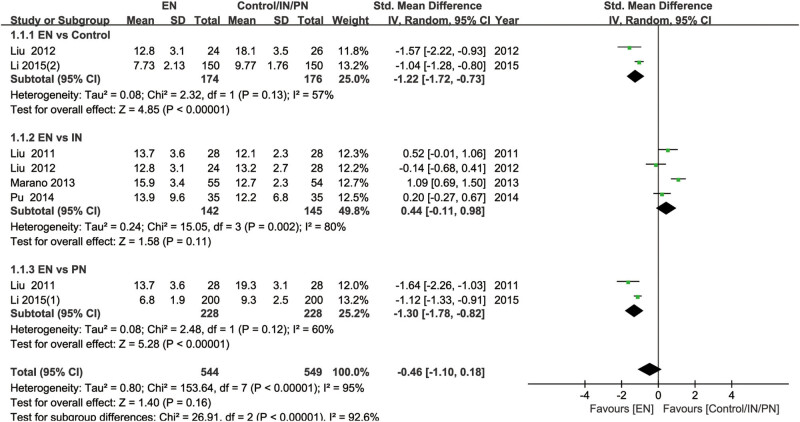
Forest plot comparing length of hospitalization in GC patients receiving EN versus those receiving IN, PN, or no nutritional intervention. GC, gastric cancer; EN, enteral nutrition; IN, immunonutrition; PN: parenteral nutrition; CI, confidence interval.

A total of six RCTs evaluated the effect of nutritional interventions on the length of hospitalization in GC patients, among which two studies comparing EN and no nutritional intervention, four studies comparing EN and IN, and two studies comparing EN and PN. As shown in Figure 3, the length of hospitalization was significantly shorter in GC patients who received EN compared with those who did not undergo nutritional intervention (SMD = −1.22, 95% CI [−1.72, −0.73], *P* < 0.001) or who received PN (SMD = −1.30, 95% CI [−1.78, −0.82], *P* < 0.001), while the benefits of EN in length of hospitalization was similar to IN (*P* > 0.05). In contrast, two RCTs investigated the influence of EN compared to no nutritional intervention on the time to first flatus in GC patients and a study reported the effect of oral nutritional supplement (ONS) compared to no nutritional intervention in GC patients. In both cases, there were no significant differences (*P* > 0.05). However, a study demonstrated that IN significantly reduced the time to first flatus compared to no nutritional intervention. In addition, four RCTs compared the effects of EN versus IN or PN on the time to first flatus. Patients receiving EN had a significantly shorter time to first flatus compared to those receiving PN (SMD = −2.14, 95% CI [−2.37, −1.91], *P* < 0.001). However, there was no significant difference compared to those receiving IN (*P* > 0.05) (Figure S1).

### 3.4 Incidence of complications

**Figure 4:**
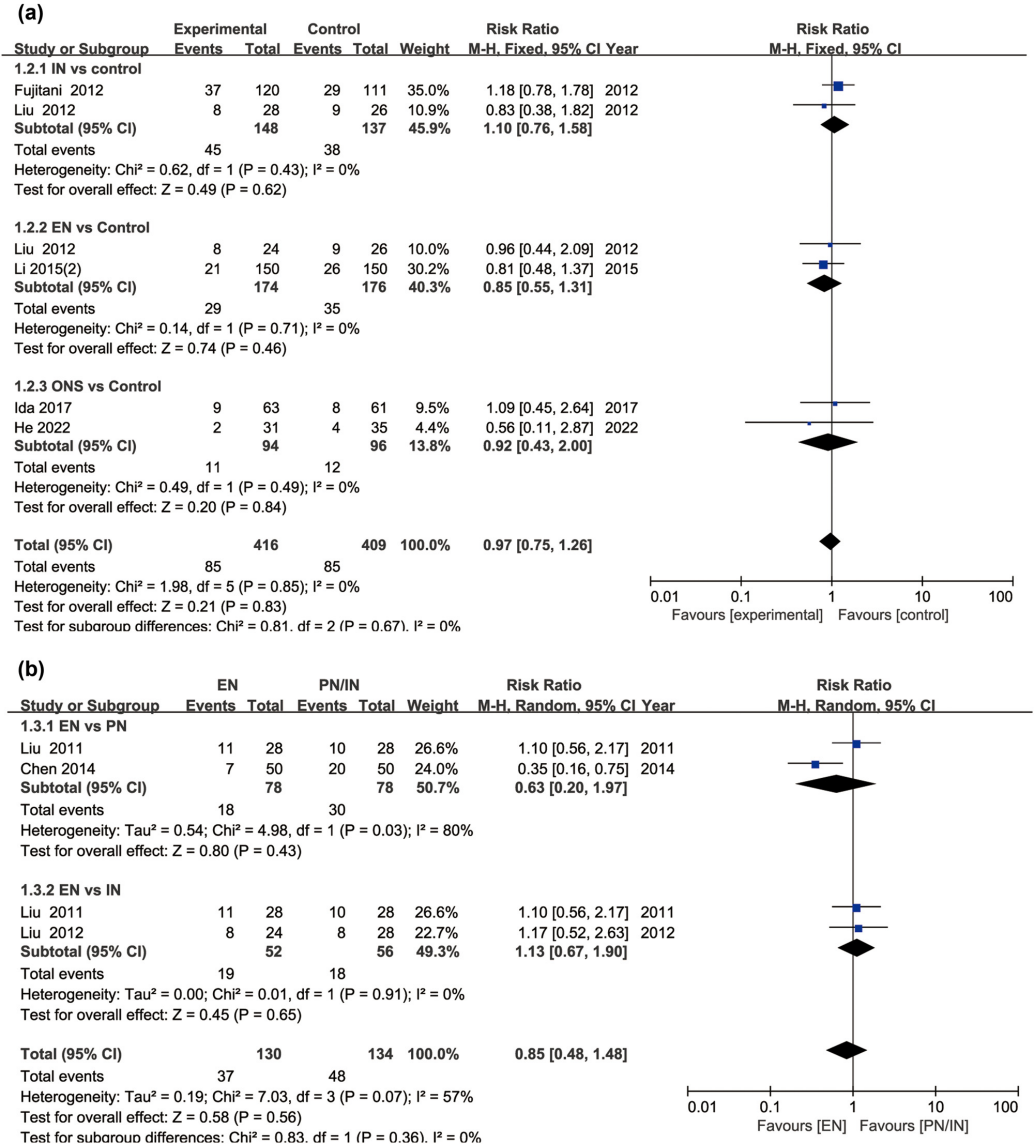
Forest plot comparing complication rates in GC patients receiving different nutritional interventions: (a) IN, EN, or ONS versus control; (b) EN versus PN or IN. GC, gastric cancer; EN, enteral nutrition; IN, immunonutrition; PN: parenteral nutrition; ONS, oral nutritional supplement; CI, confidence interval.

A pooled analysis of seven studies was conducted to evaluate whether different nutritional interventions impact complication rates in GC patients. As shown in Figure 4a, the total complication rates of IN, EN, and ONS interventions were similar to those of the control group (*P* > 0.05). In addition, there was no significant difference in total complication rates for EN compared with PN and IN (*P* > 0.05), suggesting that the risk to complication is not improved by all types of nutrition interventions (Figure 4b). Furthermore, analysis of specific complications, including gastrointestinal adverse reactions, surgical site infections, anastomotic leakage, and pulmonary infections, showed that these complication rates in patients with EN were similar to those with IN or PN (Figure S2).

### 3.5 Nutritional status

**Figure 5:**
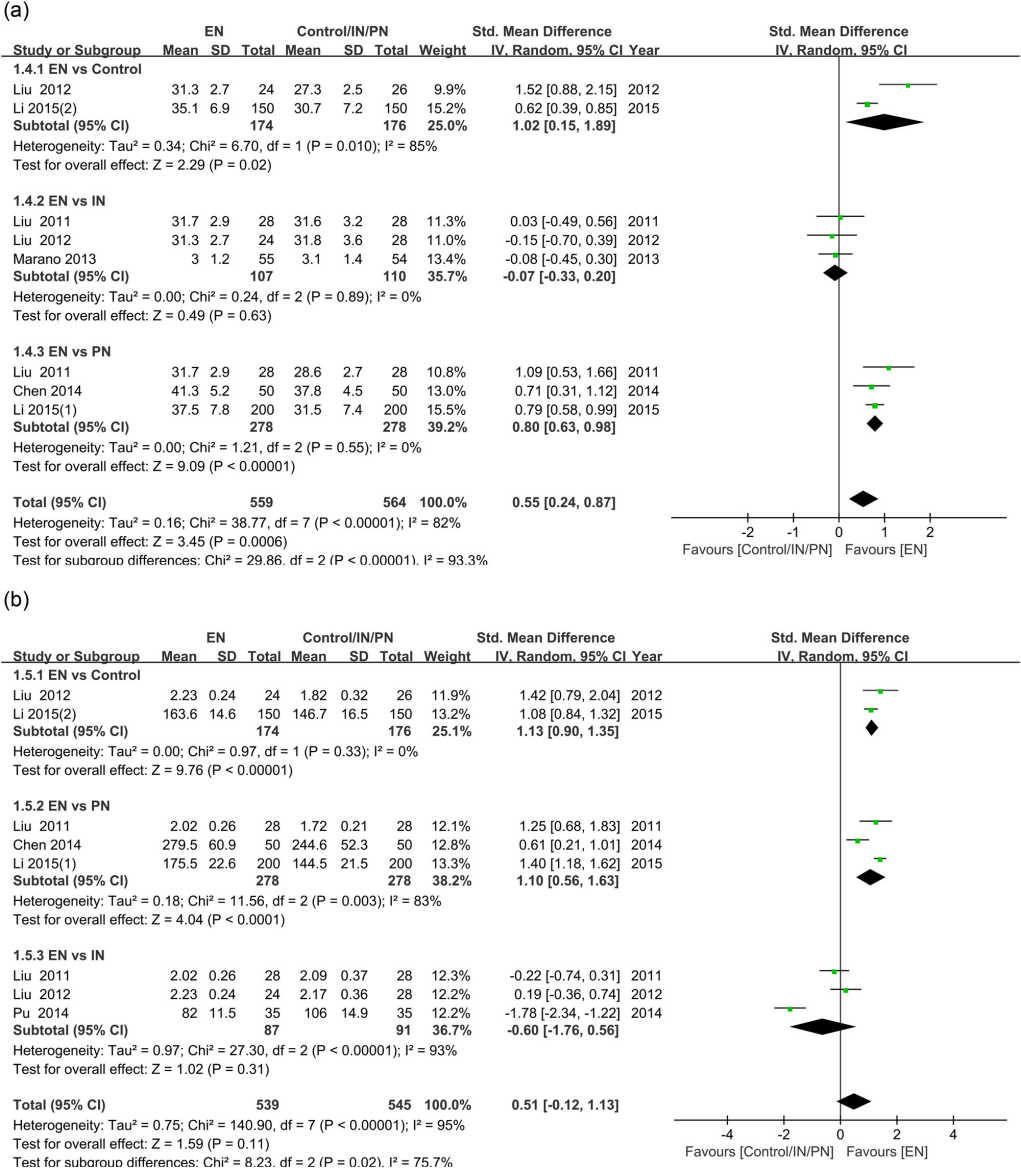
Forest plot comparing nutritional status in GC patients receiving different nutritional interventions: (a) comparison of albumin levels in EN versus control, IN, or PN; (b) comparison of prealbumin levels in EN versus control, IN, or PN. GC, gastric cancer; EN, enteral nutrition; IN, immunonutrition; PN: parenteral nutrition; CI, confidence interval.

The role of nutritional intervention on the nutritional status of GC patients was further evaluated. Both albumin and prealbumin levels were significantly higher in patients who received EN compared to patients without nutritional intervention (albumin: SMD = 1.02, 95% CI [0.15, 1.89], *P* = 0.02; prealbumin: SMD = 1.13, 95% CI [0.90, 1.35], *P* < 0.001) or who received PN (albumin: SMD = 0.80, 95% CI [0.63, 0.98], *P* < 0.001; prealbumin: SMD = 1.10, 95% CI [0.56, 1.63], *P* < 0.001), but there was no significant difference compared to IN (*P* > 0.05) (Figure 5a and b).

### 3.6 Immune function

**Figure 6:**
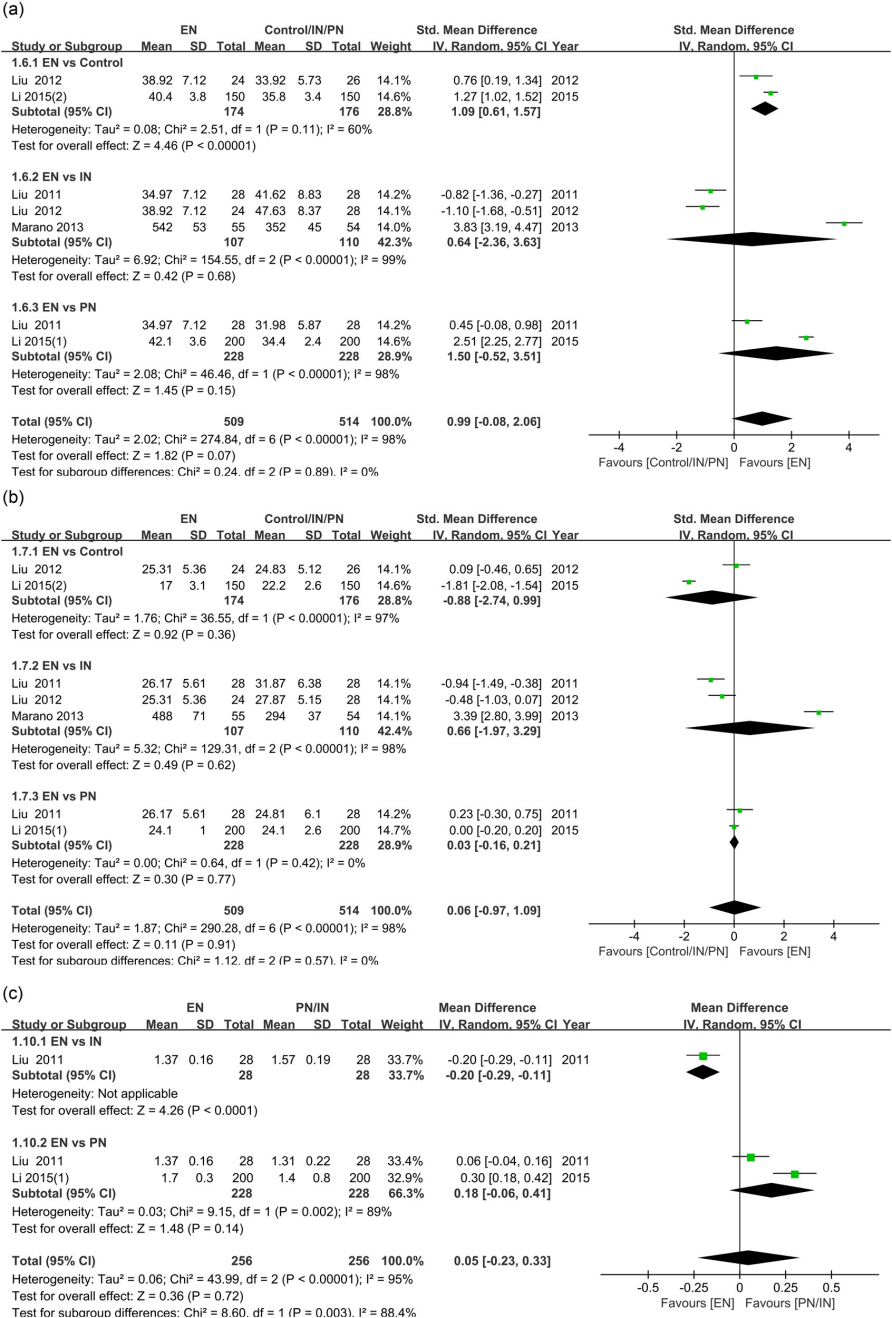
Forest plot comparing immune function in GC patients receiving different nutritional interventions: (a) comparison of CD4+ levels in EN versus control, IN, or PN; (b) comparison of CD8+ levels in EN versus control, IN, or PN; (c) comparison of CD4+/CD8+ ratio in EN versus IN or PN. GC, gastric cancer; EN, enteral nutrition; IN, immunonutrition; PN: parenteral nutrition; CI, confidence interval.

A total of five studies evaluated the effects of nutritional interventions on lymphocyte subsets in GC patients. As shown in Figure 6a, EN significantly increased CD4+ levels in GC patients compared to those without nutritional intervention (SMD = 1.09, 95% CI [0.61, 1.57], *P* < 0.001), which was similar to efficacy of IN and PN (*P* > 0.05). In addition, there was no significant difference in CD8+ levels between patients who received EN and those who received IN, PN, or no nutritional intervention (*P* > 0.05) (Figure 6b). A study reported that EN had a higher proportion of CD4+/CD8+ ratio compared to IN, and the pooled results of the two studies showed no significant difference in the CD4+/CD8+ proportion between EN and PN (*P* > 0.05) (Figure 6c). In addition, three studies compared the effects of EN and IN on immunoglobulins in GC patients, and the results showed that the IgG (SMD = −2.90, 95% CI [−3.84, −1.95], *P* < 0.001) and IgM (SMD = −0.99, 95% CI [−1.71, −0.26], *P* < 0.001) levels in patients receiving EN were significantly lower than those receiving IN (Figure S3).

### 3.7 Publication bias

**Figure 7:**
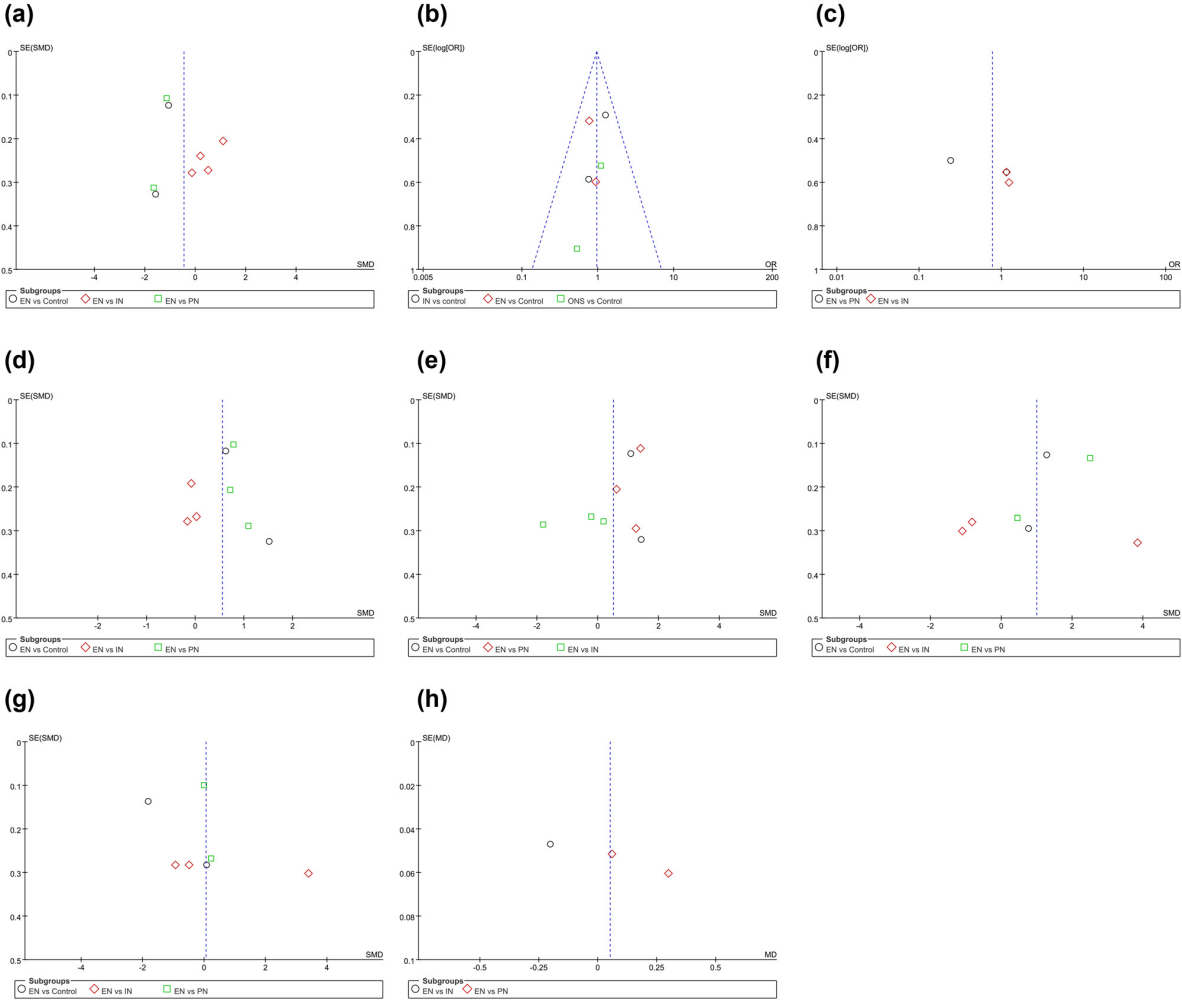
Funnel plot for publication bias test: (a) length of hospitalization, (b) complication rates in different nutritional interventions versus control, (c) complication rates in EN versus PN or IN, (d) albumin levels, (e) prealbumin levels, (f) CD4+ levels, (g) CD8+ levels, and (h) CD4+/CD8+ ratio.

Since the meta-analysis for each indicator included fewer than ten studies, we assessed publication bias using a funnel plot. As illustrated in Figure 7, most of the studies were evenly dispersed at both ends of the straight line, indicating a low publication bias.

## 4 Discussion

Due to its multifactorial pathophysiology, GC can cause different symptoms which compromise nutritional intake and metabolic balance, necessitating nutritional support [23]. Our findings suggest that nutritional support, particularly EN and IN, can significantly influence the clinical outcomes of GC patients.

There is abundant literature highlighting the advantages of early enteral feeding post-surgery, which not only enhances nutritional status but also accelerates the restoration of gastrointestinal function [11]. Previous studies have conflicting views on whether EN can shorten hospital stays compared to PN. Some argued that EN promotes faster recovery and reduces hospital stays by maintaining gut integrity and function [24], while some other studies indicated no difference in length of hospital stay between EN and PN groups [25]. In this study, EN showed a significant advantage over no nutritional intervention and PN in reducing hospital stay, while it did not exhibit a marked difference when compared with IN. However, there was a high heterogeneity in the comparison between EN and IN, which may be due to the fact that patients in EN group in Marano et al.’s research had more anastomosis leakage, leading to longer hospital stay [26]. Further investigation is needed as to whether the length of hospital stay is affected by the type of nutritional support.

EN was also shown to be superior over PN when time for first flatus, a primary indicator of postoperative gastrointestinal recovery, was considered. This is consistent with the established advantages of EN in promoting gut motility and function [27]. Similar to hospital stays, IN did not show significant differences compared to EN, which could imply that while IN has added immunological benefits, its impact on gastrointestinal recovery is comparable to standard EN.

In terms of nutritional status, our results showed the benefits of IN in elevating prealbumin levels. Prealbumin, also known as transthyretin, is a sensitive marker of nutritional status and has a shorter half-life than albumin, making it a more responsive indicator of recent nutritional changes. However, high heterogeneity was observed in both analysis of EN versus PN or IN in assessing prealbumin levels. When comparing EN group to PN group, both Liu et al. [13] and Li et al. [9] list similar composition and ratio of EN and PN nutrient solutions, but Chen and Zhang [17] offer no detail. It is uncertain whether this is the reason for the high heterogeneity. When comparing EN group to IN group, the high heterogeneity may come from different immunonutritional interventions. Pu et al. [18] used the whole protein compound preparation EN solution, while both studies of Liu et al. [13,15] added glutamine (Gln) (12.5 g/L) and arginine (9.0 g/L) to the EN solution. Besides, the significant rise in levels of albumin also suggested that the type of nutritional support might play a pivotal role. In summary, nutritional status was significantly improved in patients receiving EN compared to control and PN groups, which was similar to patients receiving IN. EN could play a pivotal role in enhancing the nutritional status of these patients, which might translate to better overall outcomes.

Our findings indicate no significant differences in overall complication rates among different nutritional interventions, supporting the safety of EN, IN, and ONS. This is in line with previous research demonstrating that nutritional support does not increase the risk of complications in surgical patients [28]. Furthermore, the analysis of specific complications, such as gastrointestinal adverse reactions, surgical site infections, anastomotic leakage, and pulmonary infections, revealed no significant differences between EN and IN or PN. This is a critical consideration, as the safety profile of nutritional interventions is paramount in clinical decision-making.

In this study, EN significantly enhanced immune function in GC patients, particularly by increasing CD4+ levels, which was crucial for postoperative recovery and infection resistance. This improvement in CD4+ levels indicate that EN supports the immune system more effectively [29]. It is noteworthy that while EN improved CD4+ levels, its efficacy was still surpassed by IN, similar to the results of CD8+ levels and CD4+/CD8+ ratio. However, there was high heterogeneity in the analysis of the effects of EN and IN on CD4+ and CD8+ levels. Among them, Marano’s study showed results at the level of CD4+ and CD8+ T cells that are contrary to two other publications by Liu et al. [13,15,26]. This might be because in Marano’s study, the IN group used a formula rich in arginine, omega-3, and ribonucleic acid, while Liu et al.’s study mainly used a formula with Gln. These components have different modes of action in immunomodulatory and anti-inflammatory responses, which could bring about a varied set of immunological outcomes. In addition, patient populations may also have differed. Two studies including Liu et al. included patients with advanced GC, while Marano et al.’s study included a broader population of patients with GC. Regarding the effects of EN and IN on immunoglobulins in GC patients, the levels of IgG and IgM were significantly lower in patients receiving EN, suggesting that IN may provide specific immunological advantages [30]. Therefore, tailoring nutritional interventions based on individual patient needs could optimize clinical outcomes, suggesting a need for further research in this domain to refine nutritional support strategies for GC patients.

Like all research, this study has its limitations. The exclusion of non-RCT studies, while strengthening the validity of our findings, might have led to the omission of valuable insights from observational studies, which often have larger sample sizes and real-world settings. Furthermore, the variability in the definition and types of nutritional support across the included studies could introduce heterogeneity, potentially affecting the generalizability of our findings. Besides, some research results need to be interpreted with caution due to the limited number and high heterogeneity of literature.

## 5 Conclusion

It is evident that nutritional interventions contribute significantly to the management and treatment of GC patients. The differential effects of IN and EN on various health outcomes underscore the need for personalized nutritional strategies tailored to individual patient needs. Future research should focus on optimizing these interventions to maximize therapeutic outcomes for GC patients.

## Supplementary Material

Supplementary material
