# Supplementary material for: Effectiveness of nutritional support for clinical outcomes in gastric cancer patients: A meta-analysis of randomized controlled trials
Source: Open Med (Wars). 2024 Sep 4;19(1):20241023. doi: 10.1515/med-2024-1023 (PMC11377983; doi:10.1515/med-2024-1023)
Supplement: Supplementary material [file med-2024-1023-sm.pdf]

Supplementary material

(a)

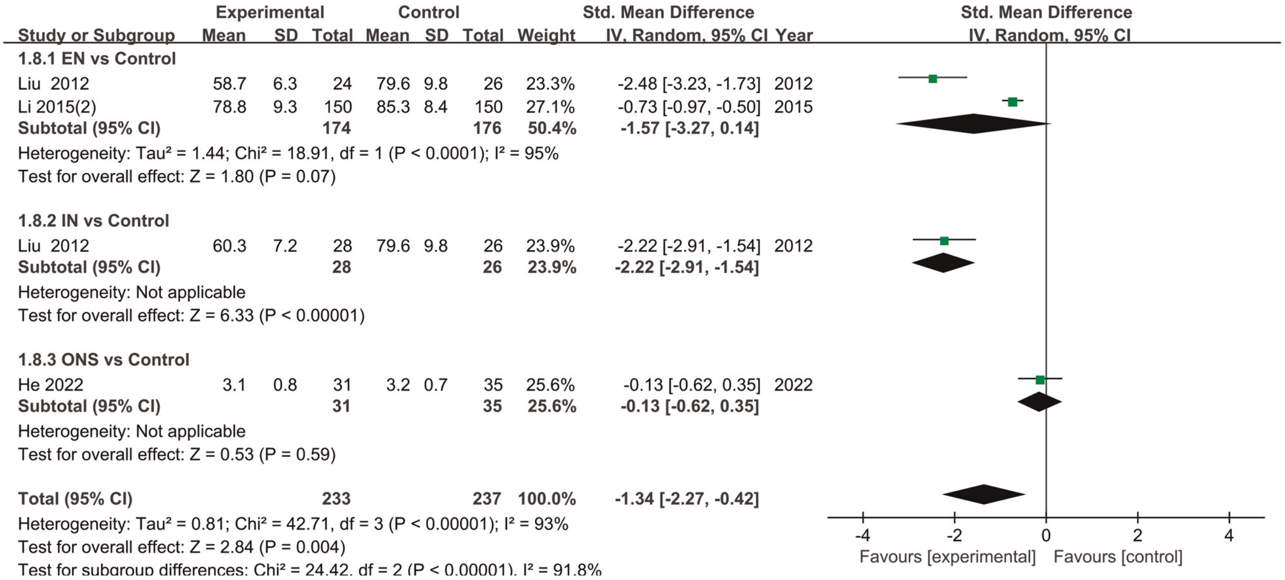

(b)

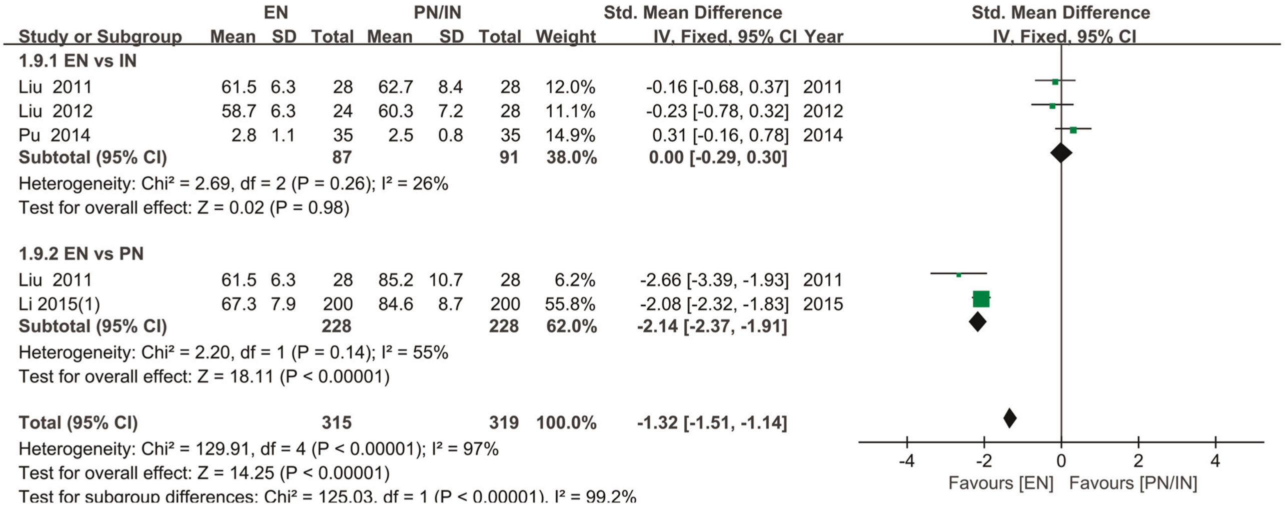

Figure S1: Forest plot comparing time to first flatus in GC patients receiving different nutritional interventions. (a) IN, EN or ONS versus control; (b) EN versus PN or IN. GC, gastric cancer; EN, enteral nutrition; IN, immunonutrition; PN: parenteral nutrition; ONS, oral nutritional supplement; CI, confidence interval.

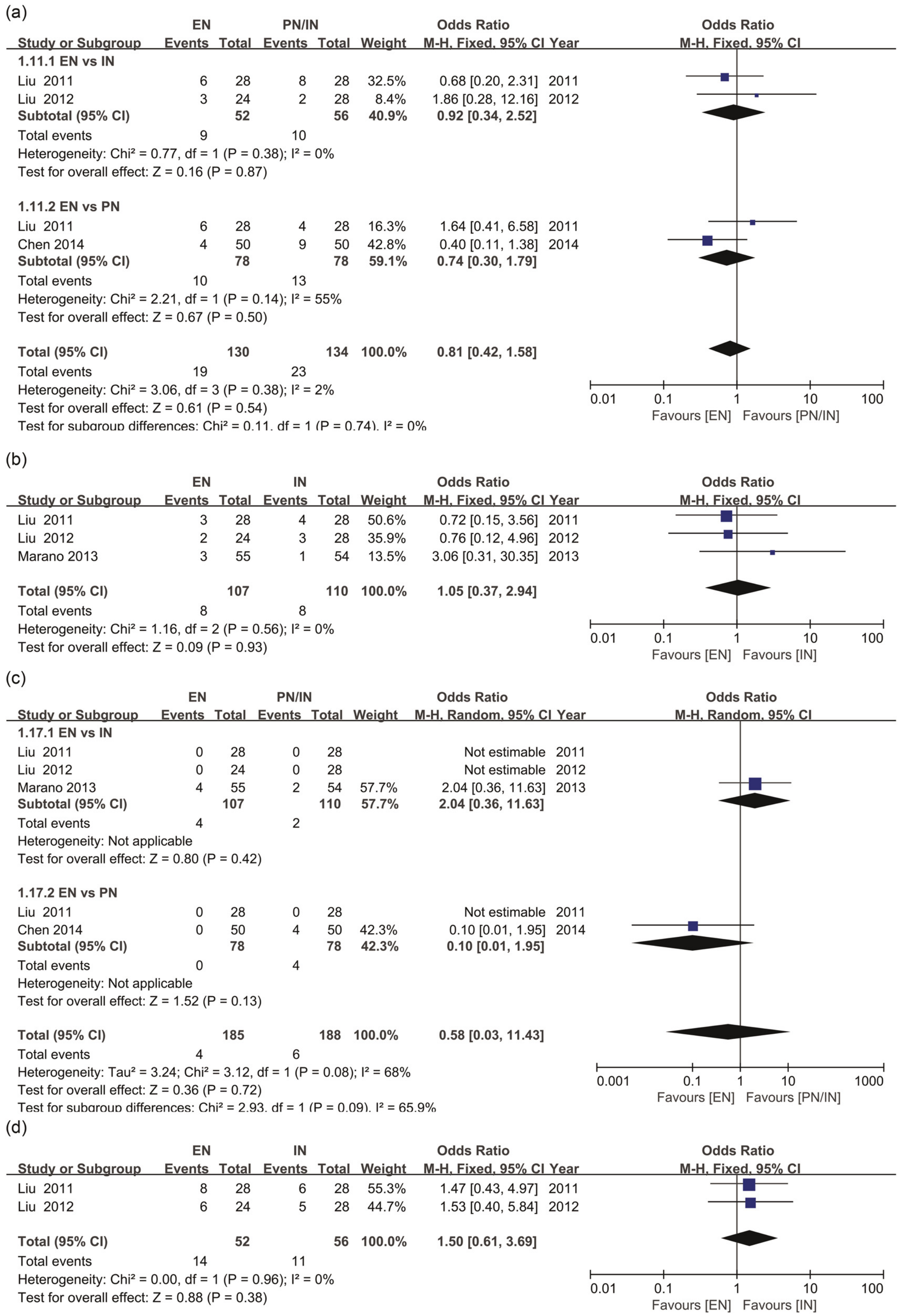

Figure S2: Forest plot comparing specific complication rates in GC patients receiving different nutritional interventions. (a) comparison of gastrointestinal adverse reactions in EN versus PN or IN; (b) comparison of surgical site infection in EN versus IN; (c) comparison of anastomotic leakage in EN versus PN or IN; (d) comparison of pulmonary infection in EN versus PN or IN. GC, gastric cancer; EN, enteral nutrition; IN, immunonutrition; PN: parenteral nutrition; CI, confidence interval.

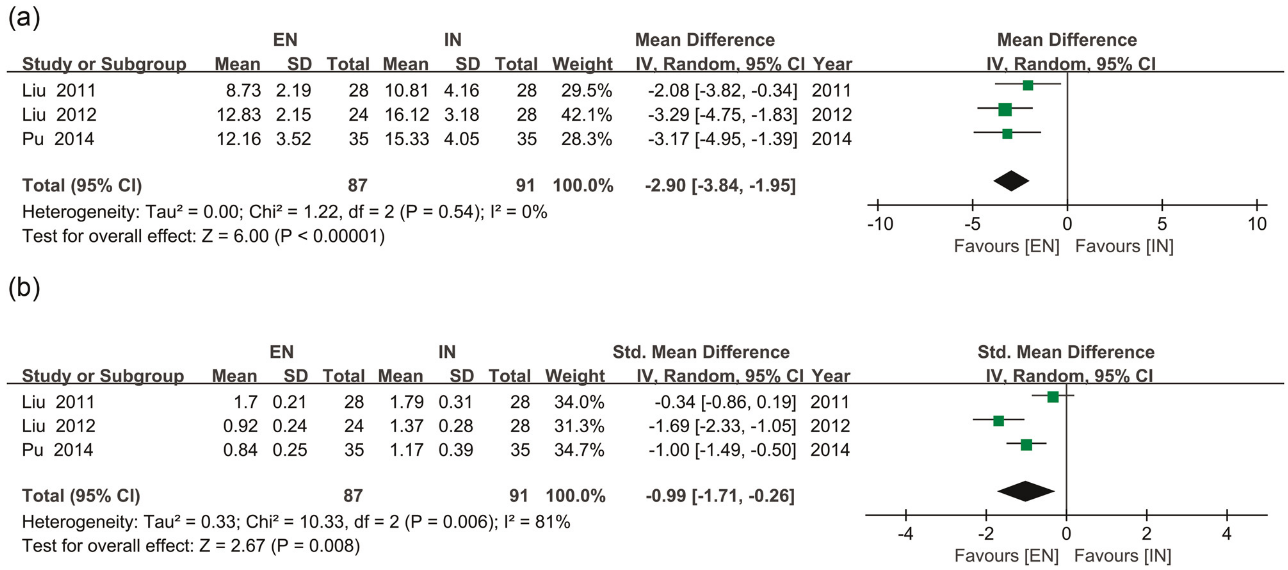

Figure S3: Forest plot comparing immunoglobulins in GC patients receiving different nutritional interventions. (a) comparison of IgG in EN versus IN; (b) comparison of IgM in EN versus IN. GC, gastric cancer; EN, enteral nutrition; IN, immunonutrition; CI, confidence interval.

Table S1: The detailed electronic search strategy

Databases	Set	Search terms	Results
PubMed	#1	“Nutrition Therapy”[Mesh] OR “Nutritional Support”[Mesh] OR (“Diet Therapy”[Mesh] OR nutri*[Title/Abstract] OR diet*[Title/Abstract] OR immunonutri*[Title/Abstract]	1131494
	#2	(“Stomach Neoplasms”[Mesh]) OR ((gastric[Title/Abstract] OR stomach[Title/Abstract]) AND (cancer[Title/Abstract] OR carcinoma[Title/Abstract] OR neoplasm*[Title/Abstract] OR oncology[Title/Abstract] OR tumour*[Title/Abstract] OR tumor*[Title/Abstract] OR malignan*[Title/Abstract]))	174500
	#3	#1 AND #2 AND [“randomized controlled trial”[pt]	343
Cochrane	#1	MeSH descriptor: [Nutrition Therapy] explode all trees	12109
	#2	MeSH descriptor: [Nutritional Support] explode all trees	4175
	#3	MeSH descriptor: [Diet Therapy] explode all trees	7749
	#4	(nutri* OR diet* OR immunonutri*):ti,ab,kw	141223
	#5	#1 OR #2 OR #3 OR #4	141351
	#6	MeSH descriptor: [Stomach Neoplasms] explode all trees	3505
	#7	(gastric OR stomach):ti,ab,kw	38822
	#8	(cancer OR carcinoma OR neoplasm* OR oncology OR tumour* OR tumor* OR malignan*):ti,ab,kw	265566
	#9	#7 AND #8	12382
	#10	#6 OR #9	12382
	#11	(randomized controlled trial):ti,ab,kw	718612
	#12	#5 AND #9 AND #10 AND #11	612
Embase	#1	'diet therapy'/exp OR 'diet therapy' OR 'nutritional support'/exp OR 'nutritional support' OR nutri*:ab,ti OR immunonutri*:ab,ti OR diet*:ab,ti	1636296
	#2	'stomach tumor'/exp OR 'stomach tumor' OR ((gastric:ti,ab OR stomach:ti,ab) AND (cancer:ti,ab OR carcinoma:ti,ab OR neoplasm*:ti,ab OR oncology:ti,ab OR tumour*:ti,ab OR tumor*:ti,ab OR malignan*:ti,ab))	273900
	#3	#1 AND #2 AND [randomized controlled trial]/lim AND ([article]/lim OR [article in press]/lim OR [preprint]/lim)	421
Web of Science	#1	(TS=(gastric OR stomach)) AND (TS=(cancer OR carcinoma OR neoplasm* OR oncology OR tumour* OR tumor* OR malignan*))	295854
	#2	TS=(nutri* OR diet* OR immunonutri*)	4205562
	#3	TS=(randomized controlled trial OR RCT)	756032
	#4	#1 AND #2 AND #3	829
